# Treatment trajectories of gender incongruent Austrian youth seeking gender-affirming hormone therapy

**DOI:** 10.3389/fendo.2024.1258495

**Published:** 2024-05-07

**Authors:** Jojo Steininger, Sarah Knaus, Ulrike Kaufmann, Johannes Ott, Stefan Riedl

**Affiliations:** ^1^ Division of Pediatric Pulmonology, Allergology and Endocrinology, Department of Pediatrics and Adolescent Medicine, Medical University of Vienna, Vienna, Austria; ^2^ Division of Gynecological Endocrinology and Reproductive Medicine, Department of Gynecology and Obstetrics, Medical University of Vienna, Vienna, Austria; ^3^ Department of Pediatrics, St. Anna Kinderspital, Medical University of Vienna, Vienna, Austria

**Keywords:** transgender, gender-affirming care, hormone therapy, legal gender marker, fertility preservation

## Abstract

**Objective:**

The aim of this study was to describe the treatment trajectories of Austrian children and adolescents with gender incongruence seeking gender-affirming medical care.

**Methods:**

Patients who presented with gender incongruence at the pediatric outpatient clinic for differences in sex development at a large university hospital in Austria from January 2008 to December 2022 were included in a retrospective chart review, and analyzed regarding referral numbers, patient characteristics, treatment trajectories, fertility preservation, and legal gender marker changes.

**Results:**

Of 310 eligible patients, 230 (74.2%) were assigned female at birth (AFAB), and 80 (25.8%) were assigned male at birth (AMAB). The number of referrals increased steeply from 2008 to 2018, whereafter it stabilized at around 50 per year. At the time of initial presentation, the median age of patients was 15.6 years (IQR 14.3-16.8). AMAB individuals tended to be younger (median 14.9 years, IQR 13.9-16.8) than AFAB individuals (median 15.8 years, IQR 14.4-16.8; p= 0.012). 207 (66,8%) completed the assessment process and were eligible for gender affirming medical treatment (GAMT). Of those, 89% (186/207) commenced gender affirming hormone therapy in the pediatric outpatient clinic (79/186 received GnRHa monotherapy, 91/186 GnRHa and sex steroids, and 16/186 sex steroid monotherapy). Of the 54 AMAB individuals receiving GAMT, 6 (11.1%) completed fertility preservation prior to therapy initiation. Only 1/132 AFAB adolescents receiving GAMT completed fertility preservation. Chest masculinization surgery was performed in 22 cases (16.7%), and breast augmentation in two cases (3.7%) between the ages of 16 and 18. Changes in legal gender marker were common, with 205 individuals (66.1%) having changed their legal gender marker.

**Conclusion:**

This is the first time that treatment trajectories, fertility preservation rates, and changes of legal gender marker have been described in Austrian adolescents with gender incongruence seeking GAMT. The majority received GAMT and changed their legal gender marker, while gender affirming surgery rates were low, and utilization of fertility preservation treatment options was rare.

## 1 Introduction

Transgender and gender diverse (TGD) individuals do not identify with their gender assigned at birth. The term TGD is used to allow the inclusion of a broad spectrum of gender identities for people who do not identify with their gender assigned at birth but might also not necessarily identify with the term “transgender” (e.g. non-binary, genderqueer, or agender individuals). An incongruence between gender assigned at birth and gender identity can lead to gender dysphoria (GD), which is associated with clinically significant distress and impairment in social, occupational, or other important areas of functioning, according to the DSM-5 (1). GD, besides being an inherently distressing experience, can also be understood within the framework of minority stress, associated with experiences of discrimination, exclusion, victimization, and internalized as well as external stigmatization (2, 3). It is thus unsurprising that GD is frequently associated with co-occurring mental health conditions such as depression, anxiety, eating disorders, self-harm, and suicidality (4–8). In order to alleviate GD, TGD persons may seek gender-affirming medical treatment (GAMT) such as hormone therapy or surgery to better align their physical appearance with their gender identity (9). Apart from GAMT, social support (such as supportive environments, social transitioning, and legal gender recognition), community involvement, psychotherapy, counselling, as well as education about and advocacy for TGD are also important strategies to decrease suffering caused by GD (10–14).

The proportion of gender diversity in the general population is difficult to assess. According to the World Professional Association for Transgender Health (WPATH) Standards of care (SOC) 8, survey-based studies of children and adolescents estimate proportions of around 1.3 % identifying as transgender (15), and 2.7 - 8,4% as TGD or gender non-conforming (16, 17). The development of secondary sex characteristics during puberty can come with significant distress for TGD adolescents (18). Gender-affirming hormone therapy (GAHT) can allow TGD adolescents to undergo pubertal development that better correlates with their gender identity. This has been shown to improve psychological well-being (19), psychosocial functioning (20), mental health outcomes (21, 22) and body satisfaction (23) in TGD adolescents. However, GAHT negatively impacts fertility (24) and potential adverse effects of long-term GAHT are not yet fully researched yet (25, 26).

GAMT for children and adolescents was established in the Netherlands towards the end of the 20th century, and most current guidelines are based on the so-called “Dutch Protocol” – a combination of gonadotropin releasing analogue (GnRHa) to suppress endogenous puberty starting as early as Tanner Stage 2-3, and gender-affirming hormone therapy with sex steroids in accordance with gender identity starting around the age of 16 (27). In Vienna, as seen in other centers worldwide, demand for GAMT in children and adolescents has increased significantly over the past decade (18, 28–30). The University Clinic for Pediatric and Adolescent Healthcare at the Vienna General Hospital began seeing gender incongruent adolescents in 2008 and has since provided consultation for over 300 children and adolescents. While research on transgender health has increased in the last years, data on the medical treatment of TGD youth is still scarce and difficult to compare, as different countries and health care systems differ in their diagnostic process, legal framework, and access to GAMT. In the European context, there is a notable paucity on data about TGD youth from central Europe. The objective of this retrospective study was to describe patient characteristics, clinical management, treatment trajectories, fertility preservation and legal gender marker changes in Austrian TGD youth.

## 2 Materials and methods

### 2.1 Study design

A retrospective chart review of all patients with gender dysphoria who presented at the pediatric endocrinology outpatient clinic for differences in sex development at the Vienna General Hospital (the only center providing gender-affirming hormone treatment for children and adolescents with gender dysphoria in the Eastern Austrian region) from January 1, 2008, to December 31, 2022, was conducted. All young individuals seeking gender-affirming care during this time were included in the review, regardless of whether gender dysphoria was formally diagnosed and/or treatment was eventually initiated or not. Individuals with non-binary gender identities were included; for the sake of clarity the terms assigned male at birth (AMAB) and assigned female at birth AFAB are used to describe the different groups. Children and adolescents with differences in sex development were excluded.

### 2.2 Diagnostic process and medical treatment protocol

The current diagnostic process and medical treatment protocol used in Vienna is in accordance with the treatment recommendations for children and adolescents with gender dysphoria, released by the Austrian Ministry of Health (31), which are based on the WPATH SOC 7 (32), and the clinical practical guideline of the endocrine society (33, 34). Children and adolescents presenting with gender incongruence are assessed individually by three different mental health professionals – a child and adolescent psychiatrist, a clinical psychologist, and a psychotherapist – as well as a pediatric endocrinologist to assess physical health and pubertal stage. The mental health assessments are frequently performed in extramural practices. If consensus regarding hormone treatment initiation is established, and there are no somatic contraindications, GAMT is initiated by the pediatric endocrinologists. Complex cases are further discussed in multiprofessional grand rounds before treatment decisions are being made. GAMT consists of the administration of GnRHa to suppress endogenous puberty, followed by, or, depending on pubertal stage, commenced simultaneously with testosterone or estrogens for transmasculine and transfeminine individuals respectively. GnRHa therapy can be commenced from Tanner stage 2-3, while testosterone and estrogens are prescribed around the age of 16 to initiate puberty of the affirmed gender (35). The GnRHa formulations used were Triptorelin s.c. or Leuprorelin s.c., depending on availability and approval. Hormone therapy for transfeminine individuals consists largely of oral estradiol formulations, while transmasculine individuals receive either intramuscular or transdermal applications of testosterone. Detailed information on the effects of gender affirming hormone therapy on fertility, as well as fertility preservation options are part of every pre-treatment discussion, and informed consent was given by every patient as well as their legal guardians prior to commencing gender-affirming hormone therapy.

### 2.3 Parameters analyzed

Age at each visit, sex assigned at birth, gender identity, and legal gender marker were recorded. Age of onset for gender dysphoria was categorized into before and during puberty. Completion of the diagnostic process (all three mental health evaluations), to be eligible for treatment were noted. Treatment start with GnRHa, as well as with sex steroids was noted. If applicable, gender affirming surgical interventions, type of intervention and age at intervention were recorded. All individuals who transitioned to the adult gender-affirming services at the university hospital were followed up until 01/2023 to identify possible changes in gender identity and further surgical interventions under the age of 20.

### 2.4 Statistical analysis

Numerical data are provided as median with interquartile range (IQR) and range (minimum to maximum) where of additional informative value, categorical parameters are provided as frequency and/or percentage. Groups were compared to each other using analyses of variance (ANOVE) for numerical parameters and chi-square/Fisher’s exact test for categorical parameters. Statistical analysis is performed using IBM SPSS Statistics Version 29.0.0.0. Statistical significance is assumed at p values <0.05. Bonferroni correction was used to correct for multiple testing.

## 3 Results

### 3.1 Patient numbers, sex assigned at birth, and gender identity

**Figure 1:**
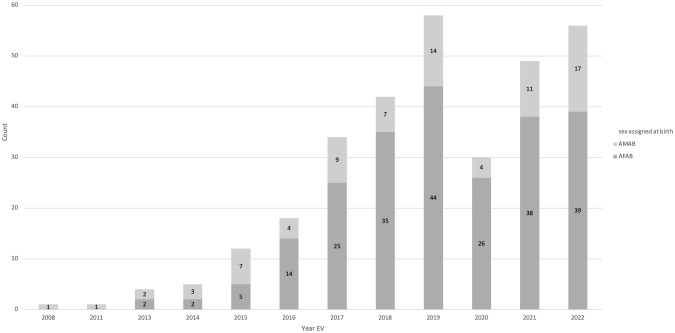
Number of initial presentations per year. In 2020, the outpatient clinic was closed for several months due to Covid-19 restrictions, leading to lower numbers that year.

In total, 310 children and adolescents with gender incongruence were included (AMAB: 80, 25.8%, AFAB: 230, 74.2%). In the years 2008 to 2015, patient numbers were very low, and the number of AMAB individuals (n=14) superseded the number of AFAB individuals (n=9) with an AMAB to AFAB ratio of approximately 1.6:1. From 2016 onwards significantly more AFABs with gender dysphoria visited our outpatient clinic, shifting the AMAB : AFAB ratio to approximately 1:3. From 2019 to 2022, the number of new referrals plateaued, with a decrease during the Covid-19 pandemic in 2020, where the outpatient clinic was closed for multiple months. The number of initial referrals per year can be seen in **Figure 1**.

The vast majority of AMAB individuals identified as transfeminine (78 transfeminine, 97.5% and 2 non-binary, 2.5%), whereas the most of the AFAB individuals identified as transmasculine (223 transmasculine, 97.0% and 7 non-binary, 3%).

### 3.2 Patient age information

The median age at first referral was 15.6 years (IQR 14.3-16.8) for the entire cohort. AMAB individuals were significantly younger (median 14.9 years, IQR 13.9-16.8, range 8.4 -17.9) than AFAB individuals (median 15.8 years, IQR 14.4-16.8, range 7.1 to 18.0; p= 0.012). Only five (2.2%) patients were below the age of 10, 26 (11.3%) were below the age of 13.

Data regarding onset of gender dysphoria were available for 282 individuals. Age of onset was early childhood and puberty in 169/282 (59.9%) and 113/282 (40.1%) individuals, respectively. Outing age to family and/or friends was recorded in 110 patients, with a median age of 13 years (IQR 12-15; range 4-17). Median age at outing was significantly higher in AMAB (14 years, IQR 14-15) than in AFAB individuals (13 years, IQR 12-15; p= 0.034).

### 3.3 Gender-affirming hormone treatment

**Figure 2:**
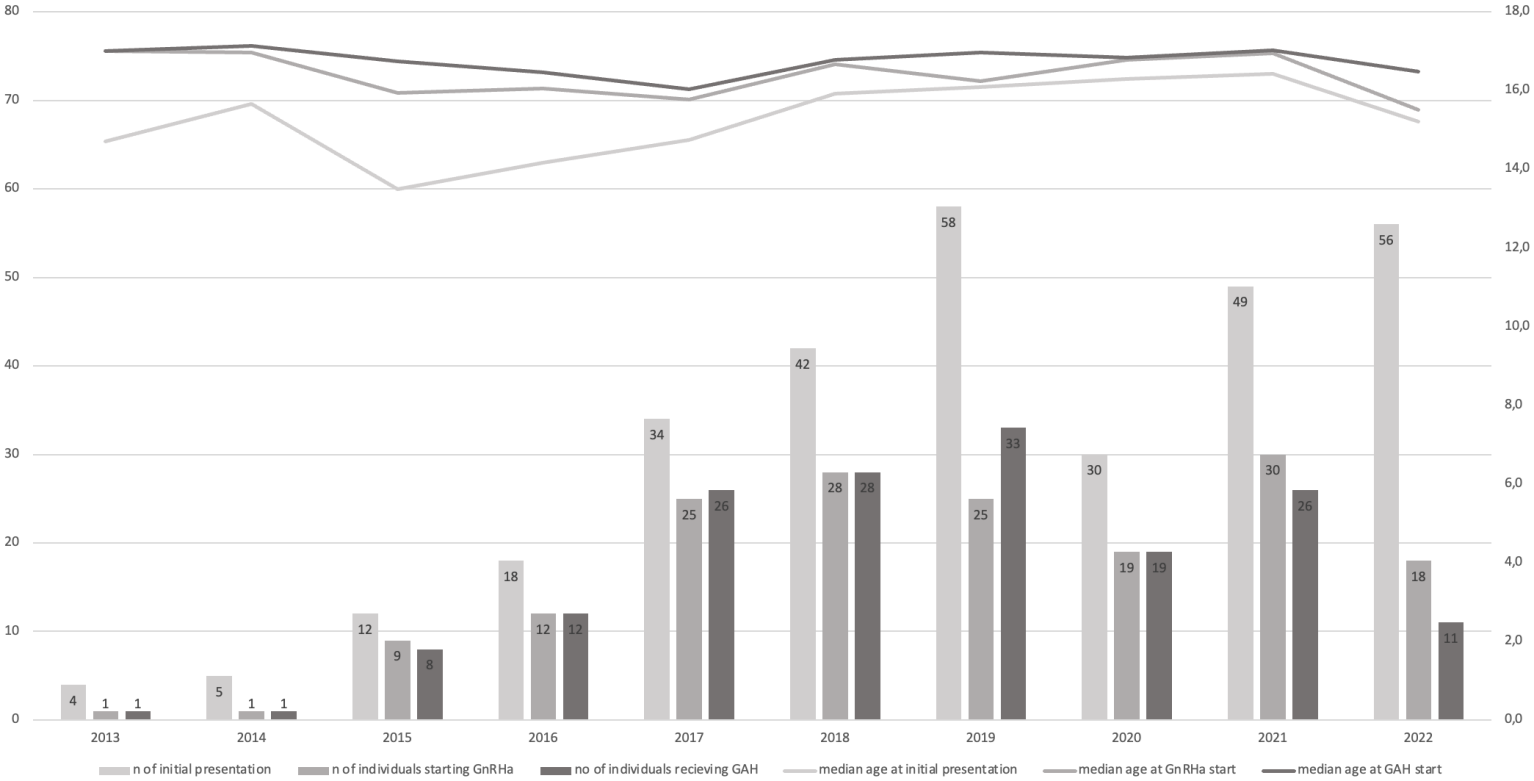
Flowchart: Hormone therapy trajectories.

Details are provided in **Figure 2**. Of the 310 TGD youth, 207 (66.8%) had completed the diagnostic process required for starting medical treatment, 48 (15.5%) had begun the diagnostic process (according to our medical files), and 55 (17.7%) did not complete any of the necessary evaluations. 89% (186/207) of the individuals who completed the diagnostic process started medical treatment in the pediatric setting – 54 AMAB and 132 AFAB individuals. The majority (91.4%, 170/186) received GnRHa. Depending on age and puberty stage, GnRHa was commenced either contemporaneously with sex steroid therapy (91/186, median age 16.8 IQR 16.2-17.4), or as monotherapy (79/186, median age 15.3 IQR 14.4 – 16.5). Most individuals receiving GnRHa monotherapy subsequently started sex steroid therapy in the pediatric setting (64/79), while 11 individuals were still receiving GnRHa monotherapy at the time of data collection, and 4 individuals were lost to follow-up. Mean GnRHa monotherapy duration was 0.6 years, with a maximum of 3.5 years. In a small group of older adolescents sex steroid therapy was commenced as monotherapy (16/186, median age 17.2 IQR 16.8-17.8). The time span between initial presentation and initiation of treatment initiation was, on average, 0.7 years.

**Figure 3:**
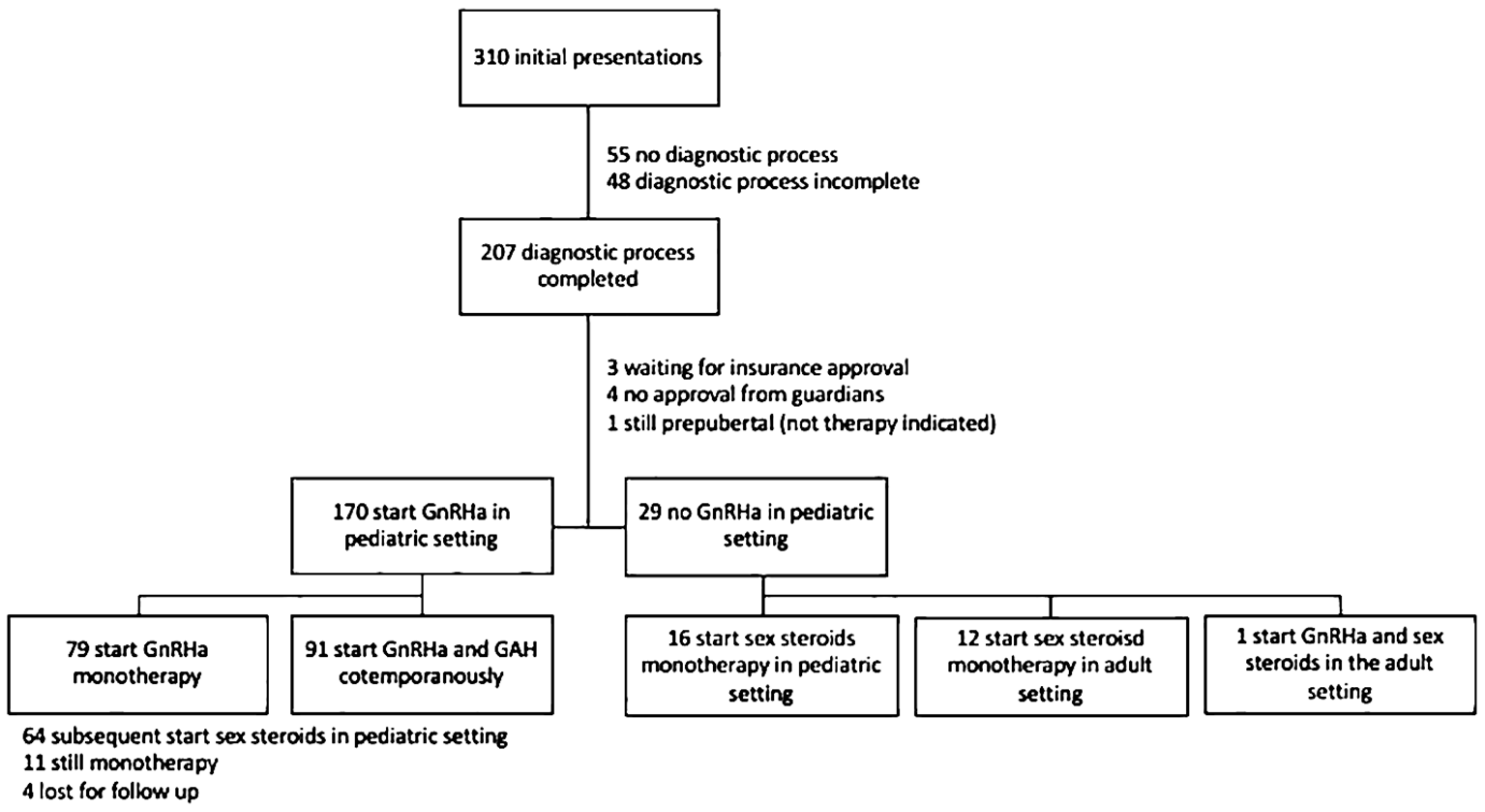
Median age at the time initial presentation and therapy initiation. Stacked bar charts presenting the number of individuals who presented initially per year, as well as the number of individuals who started GnRHa or sex steroid therapy from the number of initial presentations that year. Line charts presenting the median age at initial presentation, GnRHa treatment start and sex steroid treatment start, once again by year of initial presentation.

Trends regarding the median age at which GnRHa and hormone therapy were commenced, as well as the number of treatment utilizations grouped by year of initial presentation, can be found in **Figure 3**. Age at treatment initiation was slightly lower for AMABs compared to AFABs (median age 15.8 IQR 14.6-17.4 vs. median age 16.3 IQR 15.6-17.2), however this was not statistically significant (p = 0.062).

Notably, a further 21 individuals who began their assessment process in the pediatric setting subsequently received GAMT in the adult outpatient clinic at our center.

None of the individuals receiving GAMT discontinued treatment or expressed regret about GAMT in the pediatric setting. However, it is noteworthy that two individuals were identified who considered a detransition in the adult setting. One of these individuals discontinued GAMT temporarily, but subsequently restarted treatment. The other detransitioned. Regret regarding the initial transition was not noted in their files. Treatment trajectories of our cohort, and similar cohorts in other countries, are summarized and compared in **Table 1**.

**Table 1:** Treatment Trajectories of TGD youth in different countries.

Study	country	timespan	n total	age at first presentation	n eligible for treatment (% of all)	n receiving GnRHa (% of eligible group)	n receiving sex steroids after/with GnRHa (% of GnRHa group)	lost to follow up or discontinuation GnRHa (% of GnRHa group)
Steininger et al., 2023	AUT	2008-2022	310	14.9 AMAB 15.8 AFAB	207 (67%)	170 (82%)	155 (91%)	4 (2.3%)
Van der Loos et al, 2023 (16)	NL	1997-2018	1776	11.5 AMAB 14.1 AFAB	1401 (79%)	822 (63%)	656 (76%)	11 (0.8%)
Segev-Becker et al., 2020 (28)	Israel	2013-2018	106	15.5	96 (91%)	77 (80%)	61 (79%)	2 (2.6%)
Masic et al., 2022 (36)	UK	2017-2018	439	15.2 AMAB 14.9 AFAB	413 (94%)	401 (97%)	183 (46%)	30 (7.5%)

### 3.4 Gender affirming surgery

The most frequent gender-affirming surgery was chest masculinization surgery (“top surgery”). Of the 132 AFAB individuals receiving GAMT, 22 (16.7%) had chest masculinization surgery between the ages of 16 and 18. A further 16 individuals (12.1%) underwent top surgery shortly after reaching age of majority. Only a single AFAB patient had a hysterectomy under the age of 18. Six patients underwent hysterectomy between 18 and 20 years of age, and one transmasculine individual also received a phalloplasty before the age of 20. In AMAB individuals, breast augmentation was performed in two cases (3.7%) between the ages of 16 and 18, and two individuals (3.7%) underwent breast augmentation surgery shortly after reaching their age of majority. No surgery was performed below the age of 16. Two AMAB patients (3.7%) had vaginoplasty between the ages of 18 and 20.

### 3.5 Fertility preservation

In this cohort of TGD adolescents, six AMAB (11.1%) and one AFAB individual (0.7%) underwent complete fertility preservation (sperm and oocyte cryopreservation, respectively), prior to gender affirming treatment. Patients’ individual attitudes towards fertility preservations were not routinely documented. Of the n=61 individuals for whom this information was available, 32 stated they were not interested in having children, 14 wanted to adopt, 11 wanted children of their own (half of these ultimately underwent fertility preservation), four said they were unsure about their desire to have children, and one transfeminine individual stated her wish to experience a pregnancy.

### 3.6 Legal gender marker

The majority of TGD presenting at our clinic changed their legal gender marker (LGM) to better align with their gender identity (205/310, 66.1%). A further 14.8% (46/310) had announced their intention to do so soon. Nine individuals could not change their gender marker due to their non-Austrian citizenship (2.9%), and five individuals (1.6%) changed their first name, without changing their LGM. Notably, even amongst 124 individuals who did not receive gender affirming medical treatment in the pediatric setting, changes in LGM were common, with approximately one third having changed their gender marker, and a further third stating plans do change their gender marker soon. Details are provided in **Table 2**.

**Table 2:** Legal Gender Marker (LGM) change in TGD with and without GAMT.

	No GAMT (n=124)	GAMT (n=186)	Total (n=310)
LGM changed	44 (35.5)	161 (86.6)	205 (66.1)
Intention to change LGM	35 (28.2)	11 (5.9)	46 (14.8)
Name change without LGM change	2 (1.6)	3 (1.6)	5 (1.6)
LGM change not possible due to non-Austrian citizenship	2 (1.6)	7 (3.8)	9 (2.9)
No LGM change	41 (33.0)	4 (2.1)	45 (14.5)

Data presented in n (%).

## 4 Discussion

The present analysis describes treatment trajectories of children and adolescents who presented with gender incongruence at the pediatric clinic of Vienna University Hospital from 2008 to 2022. As in other gender clinics worldwide (18, 28–30), we observed a significant increase in the number of children and adolescents with gender incongruence and GD seeking gender-affirming care in recent years. The ratio of AMAB to AFAB individuals shifted from 1.55:1 in 2008-2015 to 1:3 in the years 2016-2022. This trend has also been observed in other gender identity clinics such as Amsterdam (18) and Valencia (37), albeit less pronounced than in Finland, where an AMAB to AFAB ratio of 1:5.2 was described in 2017 (29). A US-based study looking at gender identity data in an electronic health record database in Iowa described an AMAB to AFAB ratio of 1:5 in 12-17 year-old TGD individuals (38). A 2022 investigation based on a survey conducted across 16 states found AMAB to AFAB ratios of 1:1.5 to 1:2 in TGD US-American adolescents (39). As only a small number of pediatric TGD individuals ends up seeking GAMT, this discrepancy between data from self-report surveys and clinical records requires further investigation, and could reflect the influence of sociocultural factors on referral patterns (40).

The age at first visit (with a median of 15.8 years) at our clinic was significantly higher than in the Amsterdam cohort (11.5 years for AMABs, and 14.1 years for AFABs) (18), but similar to Israeli (28) and Finnish (29) cohorts (both 15,5 years), as well as a UK cohort with a mean age of 15.4 years (36).

Gender affirming treatment trajectories were comparable to other transgender youth cohorts. Of the eligible individuals, 89% (186/207), amounting to 60% of the entire Austrian cohort, started GAMT in the pediatric setting. In the Amsterdam cohort of Loos et. al., 63% of eligible individuals received GnRHa (18), in the Canadian cohort described Bauer et al. 62,4% of the entire study population received GAMT (41), and in the Israeli cohort of Segev-Becker et al., it was 80% (28). Comparison between different cohorts is complicated by variations in eligibility criteria for GAMT (that are at times not described in detail), as well as different health care systems and associated paths individuals must navigate to access GAMT.

No explicit regrets or treatment discontinuations of GAMT were noted in the pediatric setting, but four individuals (2.3%) who started treatment with GnRHa were lost to follow up and show no records of receiving subsequent hormone therapy in either the pediatric or the adult outpatient clinic at Vienna University Hospital. Two individuals were identified who discontinued treatment as young adults: one who detransitioned permanently, and one who considered a detransition, but subsequently restarted treatment. Feelings of regret about having received gender affirming treatment were not noted in either of their case files. The comprehensive mental health assessment conducted by three independent mental health professionals prior to therapy initiation might have contributed to the low detransition and regret rates in our cohort.

Surgery before the age of 18 was not performed routinely. The most frequent surgery was chest masculinization surgery, performed on less than a fifth of underage AFAB individuals receiving GAMT, whereas in a Dutch cohort almost 80% of eligible individuals underwent chest masculinization surgery (18). Chest masculinization surgery in adolescents is associated with significantly reduced gender dysphoria and very low regret rates (42), and has seen an increase in demand in recent years, in adults (43) as well as in adolescents (44). Low surgery rates in our cohort could be connected to relatively high age at hormone therapy initiation (median age 16.7), and the fact that at least 12 months of hormone therapy with sex steroids are recommended before gender affirming surgery (9). Another factor influencing surgery rates could be the generally reduced number of elective surgeries performed during and after the Covid-19 pandemic, as most surgeries in our cohort were performed in 2019 and 2020, with very low numbers in 2021 and 2022.

Fertility preservation rates were very low, with only six individuals undergoing cryopreservation, corresponding to five of the 42 AMAB individuals (11%) and one of the 121 AFAB individual receiving GAMT (0.8%). Low fertility preservation rates are also described in other studies (45–47), which are discordant with the reported desire for biological children in TGD individuals (24). Fertility preservation and possible effects of gender-affirming treatment on fertility are discussed routinely prior to any treatment start as part of the consent discussion, but Vienna University Hospital currently does not offer specialized fertility consultations for transgender adolescents. However, even centers offering specialized consultations still had similarly low fertility preservation rates (47). When considering these low rates, multiple factors should be considered: while the majority of TGD adolescents express a desire for future children (48.7%–67%), only a fraction is interested in having biological children (9%-35%) and most are interested in adoption (70-80%) (24). However, in an Australian Study investigating TGD parenthood, most had become parents via their partner giving birth (64%), 28% had given birth themselves, and only 5% and 3% were step- or foster parents respectively (48). To fulfill their desire for children, most TGD people have to overcome many barriers, even more so when pursuing biological offspring (24). More emphasis should be placed on improved access to fertility counseling and fertility preservation. Currently, neither procedural nor storage costs are covered by Austrian state health insurance. This creates an immense financial barrier for fertility treatment and should be addressed at a policy level to provide best possible care for all transgender children and adolescents. Insurance coverage of fertility preservation related costs might also increase fertility preservation utilization (24).

This study is, to our knowledge, the first study also reporting on changes to legal gender markers in TGD youth. Legal gender recognition is associated with improved mental health, and subsequently decreased levels of distress and suicidality (49–51). For context, it is important to note that in Austria, barriers to legal gender recognition for Austrian citizens are lower than barriers to GAMT, requiring only one mental health assessment presented to a local register office or municipal administration (52). In TGD youth, guardian approval is also necessary for legal gender recognition. Two thirds of our cohort of TGD youth changed their LGM, superseding the number of GAMT treatment initiations. While LGM changes were more common in those receiving GAMT (86.6%), more than a third of individuals who did not receive GAMT during the observational period of this study changed their LGM. This suggests that legal gender recognition can be an important aspect of affirming TGD young people’s gender identity, regardless of medical treatment initiation. More investigations into legal gender recognition in TGD youth and the impact on psychosociological wellbeing are required - especially since LGM change is a non-invasive, fully reversible measure that can aid in the alleviation of GD.

### 4.1 Limitations and considerations for further research

The main limitation of this study is its retrospective design. Not all analyzed data points could be found in all patient files. Follow-up data, especially into adulthood, was not available on all individuals. Another limitation lies in the fact that this is a single center study. Only individuals who had visited the endocrinological outpatient office were included - potentially overlooking a large number of Austrian youth seeking GAMT who had thus far only presented to mental health practitioners. Further research should focus on prospective studies investigating multidisciplinary approaches to alleviate GD, investigating treatment satisfaction, quality of life, and the mental as well as physical wellbeing of TGD children and adolescents. Measures to decrease barriers to fertility preservation and facilitate the fulfillment of TGD individuals’ desire to have children later in life - be it biologically or not - need to be addressed. The significance of legal gender recognition and other non-medical measures to decrease GD and increase overall wellbeing in pediatric TGD cohorts also bear consideration.

## 5 Conclusion

The number of transgender and gender diverse youth seeking gender affirming medical treatment in Austria increased over the last fifteen years, but has seemed to plateau between 2019 and 2022. This is the first study reporting treatment trajectories, fertility preservation and legal gender recognition rates in TGD youth in Austria. While most youth presented in middle adolescence (median age 15.8 years), GAMT rates were high, with 89% of eligible youth starting GAMT in the pediatric setting. There were no treatment discontinuations. Gender affirming surgery rates were relatively low, with chest masculinization surgery in 16.6% of AFAB individuals and breast augmentation in 3.7% of AMAB individuals. Fertility preservation rates were low. Legal gender recognition was very common in our TGD cohort, with the number of LGM changes superseding that of GAMT initiation rates. Prospective investigations into long-term treatment adherence, treatment satisfaction, somatic and mental health of children and adolescents with gender dysphoria, as well as the significance of legal gender recognition in TGD adolescents are needed.

## Data availability statement

The original contributions presented in the study are included in the article/supplementary material. Further inquiries can be directed to the corresponding author.

## Ethics statement

The studies involving humans were approved by Ethikkommission der Medizinischen Universität Wien. The studies were conducted in accordance with the local legislation and institutional requirements. Written informed consent for participation was not required from the participants or the participants’ legal guardians/next of kin in accordance with the national legislation and institutional requirements.

## Author contributions

JS: Conceptualization, Data curation, Investigation, Visualization, Writing – original draft. SK: Conceptualization, Data curation, Formal 1nalysis, Project administration, Writing – review & editing. UK: Data curation, Writing – review & editing. JO: Software, Supervision, Validation, Writing – original draft, Writing – review & editing. SR: Conceptualization, Methodology, Writing – review & editing.
